# Air to Tuberculosis

**Published:** 1913-12

**Authors:** Charles D. Walcott

**Affiliations:** Secretary, Smithsonian Institution


### ANNOUNCEMENT OF AWARD BY THE SMITHSONIAN INSTITUTION OF HODGKINS PRIZE FOR ESSAY "ON THE RELATION OF ATMOSPHERIC AIR TO TUBERCULOSIS."

On the recommendation of the Committee on the Award of the Hodgkins Prize of $1,500.00 for the best treatise "On the Relation of Atmospheric Air to Tuberculosis," which was offered by the Smithsonian Institution in connection with the International Congress on Tuberculosis held in Washington in 1908, the Institution announces that the prize has been equally divided between Dr. Guy Hinsdale of Hot Springs, Virginia, for his paper on "Tuberculosis in Relation to Atmospheric Air", and Dr. S. Adolphus Knopf of New York City, for his treatise "On the Relation of Atmospheric Air to Tuberculosis".

The members of the Committee on Award were:

Dr. William H. Welch, Johns Hopkins University, Baltimore, Maryland, Chairman; Dr. Hermann M. Biggs, New York City, Prof. W. M. Davis, Cambridge, Mass.; Dr. G. Dock, Washington University Medical School, St. Louis Mo.; Dr. Simon Flexner, Rockefeller Institute for Medical Research, New York City; Dr. John S. Fulton, Baltimore, Maryland; Brig. Gen. George M. Sternberg, U. S. A., (Retired), Washington, D. C.

CHARLES D. WALCOTT,
Secretary, Smithsonian Institution.

